# Clinical Relevance of Cervical Kinematic Quality Parameters in Planar Movement

**DOI:** 10.1111/os.12435

**Published:** 2019-03-18

**Authors:** Ze Guo, Wei Cui, Da‐cheng Sang, Hong‐peng Sang, Bao‐ge Liu

**Affiliations:** ^1^ Department of Orthopaedic Surgery Beijing Tiantan Hospital, Capital Medical University, China National Clinical Research Center for Neurological Diseases Beijing China

**Keywords:** Cervical spine, Center of rotation, Instantaneous center of rotation, Instantaneous axis of rotation, Kinematics, Planar movement

## Abstract

Comprehending cervical spinal motion underlies the understanding of the mechanisms of cervical disorders. We aimed to better define the clinical relevance of cervical spine kinematics, focusing on quality parameters describing cervical spine planar motion. The most common study focuses were kinematic quality parameters after cervical arthroplasty and in normal subjects, patients with cervical degeneration, and patients with cervical deformities. Kinematic quality parameters are important for cervical degeneration prevention, being detected sooner than differences on imaging examinations and being significantly related to the degree of cervical degeneration. Kinematic quality parameters are effective for evaluating the changes of cervical motion pattern after cervical fusion and non‐fusion, assessing operative and adjacent segments in the early stages, and predicting adjacent segment degeneration. However, owing to current research limitations, and controversy about the changes of kinematic quality parameters after different surgical procedures, current assessments are limited to cervical spine flexion and extension. Different osteotomy methods of cervical deformity have different effects on cervical motion patterns and quality parameters. Choosing the most effective surgical method remains a challenge and kinematic quality parameters in cervical deformity are important future research topics. This review highlights the instantaneous center of rotation, the center of rotation, and the instantaneous axis of rotation as being important kinematic quality parameters of cervical spinal motion. These can be used to detect abnormal cervical mobility, to diagnose cervical degeneration, to design disc protheses, and to evaluate surgical effects earlier than other methods. Owing to limitations of research methods there is variation in the way parameters are defined by various researchers. No uniform standard exists for defining degenerative motion quality parameters in normal asymptomatic, degenerative, and postoperative patients. Therefore, further study is required. New study techniques and defining kinematic quality parameters in normal subjects will clarify the definitions of these parameters, enhancing their future clinical usefulness.

## Introduction

Range of motion (ROM) is widely accepted as a kinematic parameter for evaluating the quantity of cervical motion. However, abnormal patterns of motion can still be present within individual segments with a normal ROM^1^. Abnormalities of the cervical spine could be revealed by the abnormal quality of motion within individual segments^1^, ^2^. The variations of the kinematic parameters, including the instantaneous center of rotation, the center of rotation, and the instantaneous axis of rotation can be used to assess the quality of cervical motion earlier than ROM^1^, ^2^.

In terms of quality in assessing cervical motion, the quality parameters are more sensitive in detecting abnormal cervical mobility resulting from cervical disorders^3^, ^4^, ^5^, ^6^. Currently, the kinematic quality parameters can be used to detect abnormal cervical mobility, to assist in diagnosis of cervical degeneration, and to evaluate surgical effects. Therefore, understanding the quality of intact cervical spinal motion is the basis for understanding the mechanisms of cervical disorders, which may improve surgical outcomes and aid in the design of disc prostheses. Accordingly, we review the recent advances in cervical spine kinematics and then clarify the clinical correlation between parameters of motion quality and the cervical spine.

**Figure 1:**
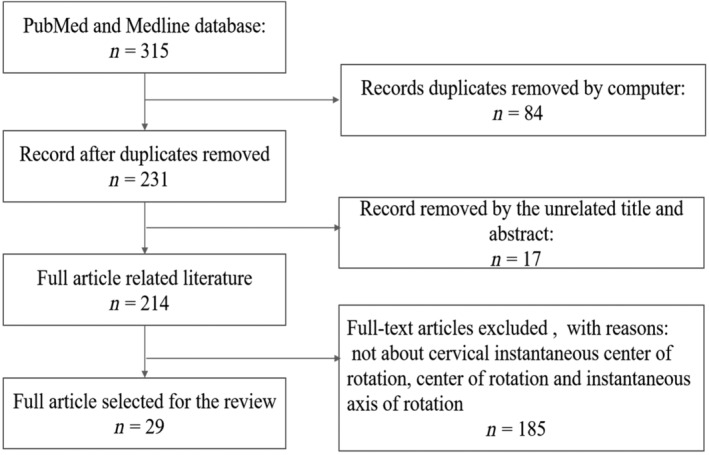
Flow chart of the search for published reports showing the process of inclusion and exclusion.

PubMed and MEDLINE database searches of articles published in English up tp 2018 using the terms “cervical spine,” “kinematic,” “instantaneous center of rotation,” “center of rotation,” and “instantaneous axis of rotation” returned 29 studies. Studies were selected if they met the following three inclusion criteria: (i) clinical and basic research on the cervical kinematic parameters including the instantaneous center of rotation (ICR), the center of rotation (COR), and the instantaneous axis of rotation (IAR); (ii) studies including normal subjects, patients with cervical degeneration or cervical deformity, and postoperative cervical patients. The exclusion criteria included the following: (i) incomplete data; (ii) patients with injuries to the cervical spine; (iii) reviews, meta‐analyses, letters to editors, abstracts, editorials, and commentaries. In this study, articles were limited to those written in the English language. The search process was performed as presented in Fig. 1.

**Figure 2:**
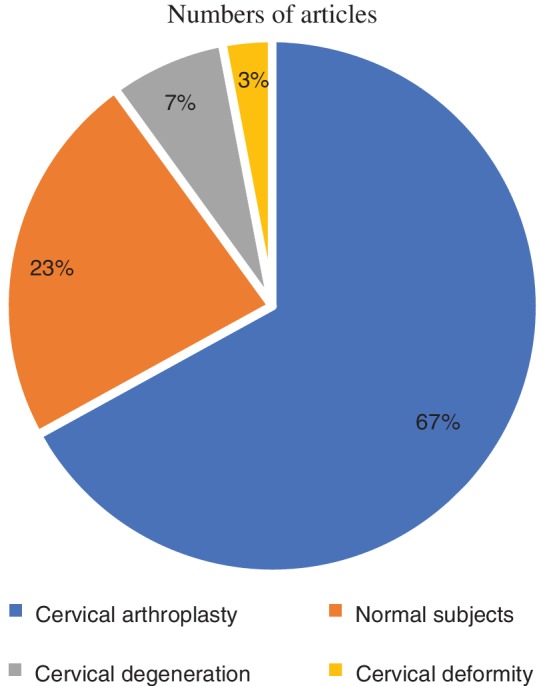
Proportion of types of articles reporting kinematic quality parameters associated with the cervical spine: the largest number of studies were associated with cervical arthroplasty, followed by normal subjects, and the smallest number of studies were associated with cervical degeneration and cervical deformity.

The finial PubMed search returned 29 studies. A flow chart of the screening process is shown in Fig. 1. The most common study focuses were kinematic quality parameters after cervical arthroplasty (*n* = 20), followed by kinematic quality parameters in normal subjects (*n* = 7), patients with cervical degeneration (*n* = 2), and patients with cervical deformities (*n* = 1). There was 1 repetitive article between the normal subjects and the cervical degeneration patients (Fig. 2).

### *Definitions of Instantaneous Center of Rotation*

**Figure 3:**
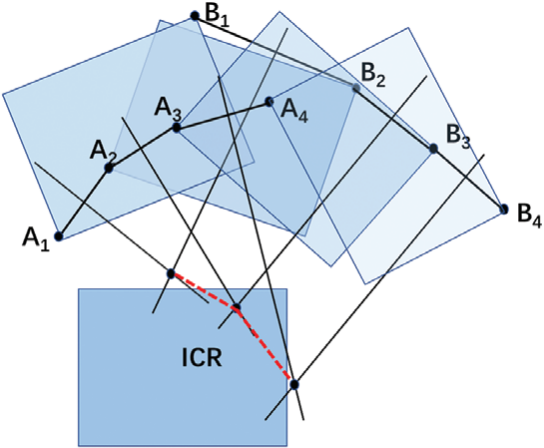
The instantaneous center of rotation paths during flexion–extension in the cervical spine: the trajectory of the vertebral body at each moment can be regarded as an arc centered on a point, which is called the instantaneous rotation center, and it is a group of points.

**Figure 4:**
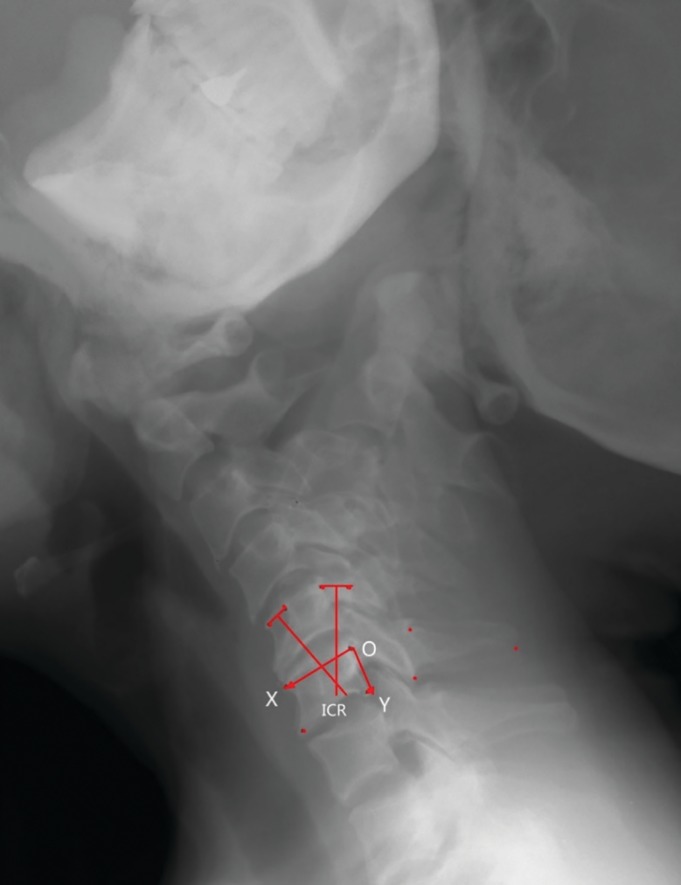
Measurement of the instantaneous center of rotation in flexion–extension in a plain lateral X‐ray by superimposing the underlying cervical vertebra according to the perpendicular bisectors and establishing the coordinates (From Liu *et al.* 3).

Instantaneous center of rotation (ICR): The instantaneous center of rotation is defined as the point with zero velocity in the plane of movement of a rigid body^7^. The instantaneous center of rotation is mainly applied to the study of spinal kinematics. The motion trajectory of the vertebral body at each moment can be regarded as a circular arc centered at a certain point, which is referred to as the instantaneous center of rotation. Thus, the instantaneous center of rotation is a cluster of trajectories composed of a group of points (Fig. 3)^7^. The location of the points can be determined using a simple geometric method: using lateral X‐rays of the cervical spine obtained under flexion and extension conditions. The lower vertebral body on the X‐ray image can be superimposed at two moments, and a line can be drawn to connect the points of two moving upper vertebral bodies. The vertical bisector intersection point at the location is determined as the instantaneous center of rotation^2^. Our team^3^ used Mimics 16.0 software to automatically superimpose two X‐ray images of the motion position to reduce human error. The corticomedullary junction is, as indicated by the outline of the vertebral body and the attachment, the turning point of the outline of the vertebral body, and the attachment is marked as an image landmark. Two midlines are drawn: the midline perpendicular to the anterior edge of the upper vertebral body and the midline perpendicular to the posterior edge of the vertebral body. The intersection of the two midlines is located at the instantaneous center of rotation (Figs 3 and 4).

### *Definitions of Center of Rotation*

**Figure 5:**
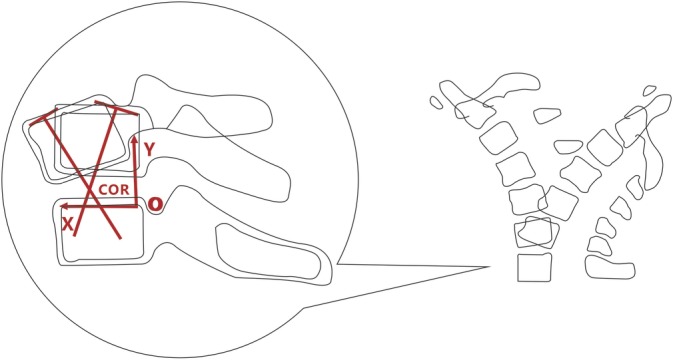
Technique of reconstructing the center of rotation: It is determined by the intersection of the vertical bisectors of the two positions on the vertebral body at two moments.

Center of rotation (COR): The center of rotation is also generally determined by the intersection of the perpendicular bisector of two points on the vertebral body during complete flexion and complete extension of the cervical spine (Fig. 5)^8^. Center of rotations comprise a group of data obtained by measuring a certain number of patients, and then under this premise, a set of points describing the location and regional distribution are obtained.

### *Definitions of Instantaneous Axis of Rotation*

**Figure 6:**
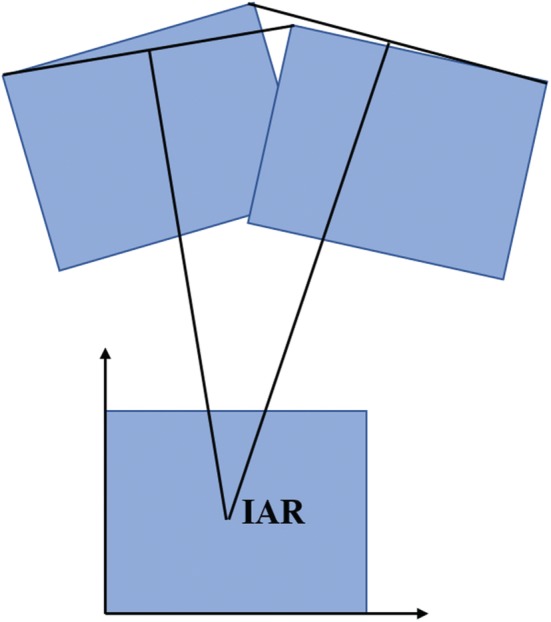
Instantaneous axis of rotation: the location of the instantaneous axis of rotation can be expressed in digital coordinates in relation to X and Y axes constructed tangential to the inferior and posterior borders of the lower vertebra.

Instantaneous axis of rotation (IAR): For a rigid body moving in a plane, at any instant, there is a line inside the body or an imaginary extension line that does not move. This line is perpendicular to the plane of rigid body motion and passes through the instantaneous center of rotation^7^, ^12^, ^16^. Currently, confirming the location of the instantaneous axis of rotation is primarily achieved by first determining the location of the instantaneous center of rotation, then passing through the instantaneous center of rotation, and, finally, determining a line that is perpendicular to the plane of motion of the cervical spine^16^ (Fig. 6).

Our goal was to define the clinical relevance of cervical spine kinematics. The instantaneous center of rotation, the center of rotation and the instantaneous axis of rotation are quality parameters that can describe the plane motion of the cervical spine. However, strict differences exist in the definitions of these parameters. The method of describing the position is the same, but the center of rotation is understood to be a unique point of the instantaneous center of rotation trajectory and is not a cluster of trajectories. Considering the technical difficulty of calculating the trajectory of the instantaneous center of rotations from cervical flexion to extension, most researchers use additional points at different times to measure the location and distribution of the instantaneous center of rotations. The instantaneous axis of rotation is a line, but in some studies that use the instantaneous axis of rotation, it is still considered as a point and is similar to the instantaneous center of rotation^13^, ^14^. Therefore, with the improvement of research techniques and methods, the definitions of the quality parameters will become clearer.

## Location of Kinematic Quality Parameters in Normal Asymptomatic Subjects

Cervical arthroplasty has been performed as an operation for treating cervical spondylosis with the aim of preserving motion^9^. Although many disc prostheses are designed to preserve motion, the kinematic properties of the currently available prostheses differ^10^. Therefore, understanding the normal cervical kinematics is fundamental for understanding the mechanisms of spinal disorders, improving clinical treatment, and designing disc prostheses.

The location of the instantaneous center of rotation in non‐degenerative cervical spines was first described by Penning^15^. By measuring the instantaneous center of rotation of 20 young adult volunteers at two moments of flexion and extension, the distribution of the instantaneous center of rotation in a group of normal people was obtained. The locations of the instantaneous center of rotation in different segments were different. In each segment of the lower cervical spine, the instantaneous center of rotation was located near the intervertebral disc, and the instantaneous center of rotation in the higher segment was located at the lower position. However, Penning did not provide any statistical parameters, such as average position and variance, although the distribution of data was shown graphically. In addition, the way in which instantaneous center of rotations with different vertebral sizes are drawn on a single common contour of the cervical spine, which requires some form of standardization, was not described. Dvorak *et al.* 11 studied 22 women (age range 25–49 years, average 30.9 years) and 22 men (age range 23–42 years, average 31.6 years), all healthy and asymptomatic, who underwent passive flexion/extension examinations of the cervical spine. Functional X‐rays were taken and analyzed using a computer assisted method that quantified locations of the centers of rotation for each level from C_l_–C_2_ to C_6_–C_7_. From C_2_–C_3_ to C_6_–C_7_, the COR move anterior–superior for each successive segment. The results were similar to those reported by Penning^15^.

Amevo^16^ studied 46 volunteers aged 22–66 years with no history of cervical pain, cervical spondylosis, neck trauma or pain caused by head and neck movement, who were regarded as the normal cervical spondylosis population. The location of the instantaneous center of rotation was measured by the vertical intersection method on cervical hyperextension, flexion and lateral X‐rays, and the ratio of vertebral height to width was used as the criterion. The instantaneous center of rotation was found below the intervertebral disc and posterior to the center of the endplate. Nevertheless, due to measurement errors and coordinate system changes, this instantaneous center of rotation data has not been widely accepted as an accurate standard by other researchers.

Anderst *et al.* 17 first calculated the motion path of the instantaneous center of rotation during dynamic motion between adjacent vertebral bodies in 20 asymptomatic controls (7 males, 13 females, average age 46 ± 6 years) using biplane radiographs. The author found that the instantaneous center of rotation between adjacent vertebrae in asymptomatic control subjects was generally fixed in the superior–inferior direction, but it translated in the anterior–posterior direction during flexion–extension. The instantaneous center of rotation in the superior–inferior direction was located near the center of C_3_ for C_2_–C_3_ and moved progressively superior (closer to the intervertebral disc) for each motion segment until C_6_–C_7_, where the instantaneous center of rotation was located near the top endplate of C_7_. Therefore, disc replacements replicating asymptomatic in vivo cervical motion should account for level‐specific differences in the location and motion path of the instantaneous center of rotation.

Jonas *et al.* 18 investigated the instantaneous center of rotation in cadaveric specimens and found that the instantaneous center of rotation in the C_2__–3_, C_4–5_ and C_6–7_ segments was located either within the caudal endplate of the respective disc or below it, which was different from the instantaneous center of rotation pathway described by Anderst^17^.

The “natural” change in the instantaneous center of rotation resulted from physiologic aging and normal degenerative changes, which should not be defined as abnormal parameters. Liu *et al.* 3 studied the cervical X‐rays of 680 asymptomatic subjects (363 males and 317 females, aged between 20 and 79 years), and they found that in asymptomatic subjects, the instantaneous center of rotation was located approximately at the superior half of the lower vertebral body height and the posterior half of its width, as well as that the instantaneous center of rotation at C_5,6_ level was more anterior and higher in patients over 50 years of age (*P* < 0.05). These findings should be considered in clinical practice and when designing disc prostheses.

In summary, most of the data showed that the locations of kinematic quality parameters gradually approach the intervertebral disc, moving progressively inferior from C_2_–C_3_ to C_6_–C_7_. However, no uniform standard exists for the location of the cervical kinematic quality parameters in normal subjects because the research techniques and objects are not standardized. Due to the limitations of research techniques, most included studies often reported combinations of the instantaneous center of rotation, the center of rotation, and the instantaneous axis of rotation. Therefore, developing new study techniques and calculating the location of kinematic quality parameters in normal subjects are topics of great significance to guide the determination of abnormal quality parameters. Our team used robotic technology to continuously measure the instantaneous center of rotation location from C_3_ to C_7_ in 11 cadavers. We found that the instantaneous center of rotation becomes more widely distributed when moving inferiorly from C_3–4_ to C_6–7_. This finding should be considered in clinical practice when designing disc prostheses.

## Clinical Application of Kinematic Quality Parameters in Cervical Degeneration

The present study quantitatively assessed the impact of cervical disc degeneration on the location of the quantitative parameters. There is a correlation between the location of the instantaneous center of rotation and the age‐related degeneration in asymptomatic subjects^3^. A displaced center of rotation could be related to changes in the movement strategy due to pain, coordination problems or a decreased movement range. Determination of the instantaneous center of rotation may be useful in diagnosing deviations of normal segmental motion in the sagittal plane or in assessing therapy in the neck region^19^. Thus, determining the instantaneous center of rotation should be considered in early clinical diagnosis and especially when designing disc prostheses.

The degeneration affected the location of the instantaneous axis of rotation^22^. Cervical degeneration can lead to structural changes in bone and soft tissue, such as the growth of osteophytes, a reduction of disc height, a decrease in ligament elasticity, an increase in facet joint pressure, the instability in the spine, and, ultimately, a change in spinal activity and the location of the quality parameters. Liu *et al.* 3 studied the cervical X‐ray of 680 asymptomatic subjects (363 males and 317 females, aged between 20 and 79 years) divided into six 10‐year age groups. The instantaneous center of rotations from C_3,4_ to C_6,7_ were determined from the radiographs using MIMICS software. A scoring system determined from lateral radiographs quantitatively assessed the degeneration of cervical intervertebral discs. Instantaneous center of rotations were compared between groups to analyze age‐related changes and the relationship between degenerative changes and instantaneous center of rotation location. The authors found that the position of the instantaneous center of rotation changed with age. In the degenerative group, the change of the instantaneous center of rotation was significantly correlated with age. The average forward displacement of the instantaneous center of rotation was greater in the moderate and severe degenerative groups than in the normal group. The loss of intervertebral disc height was the main factor leading to the change of instantaneous center of rotation with intervertebral disc degeneration.

Thus, kinematic quality parameters are important for the prevention of cervical disease development. However, due to the limitations of research methods, there is no unified standard for describing the location and distribution of degenerative motion quality parameters. Our team found that the location of the instantaneous center of rotation varied with increasing age^3^; therefore, different age groups should have different standards for kinematic quality parameters. Abnormal quality parameters can be detected earlier than differences on imaging examinations and are significantly related to the degree of cervical degeneration^3^. The instantaneous center of rotation, the center of rotation, and the instantaneous axis of rotation could be used to detect various types of cervical degeneration in the future.

## Clinical Application of Kinematic Quality Parameters in Different Operations for Cervical Degeneration

Disc replacements have either fixed or variable centers of rotation, and it is unclear how well these designs mimic in vivo cervical spine movement. In addition, an abnormal motion path of the instantaneous center of rotation in motion segments adjacent to arthrodesis may reflect altered adjacent segment loading, potentially leading to adjacent segment degeneration.

Studies have demonstrated that the location of quality parameters in artificial discs and anterior cervical decompression and fusion (ACDF) affect cervical biomechanics^31^, ^32^, ^33^, ^34^, ^35^, ^36^, ^37^, ^38^, ^39^, ^40^. ACDF is a classic operation for cervical spondylosis. Cervical arthroplasty is the current standard option utilizing nonfusion technology. Different surgical methods will affect the kinematic characteristics of the surgical segments and adjacent segment of the cervical spine. The change in the center of rotation in adjacent segments postoperatively will change the stress of the intervertebral disc, which may lead to degeneration^23^. Adjacent segment degeneration requiring reoperation has been documented at a rate of 2.9% annually after anterior cervical arthrodesis for the treatment of cervical spondylosis by Hilibrand *et al.* 24. However, the retention of the kinematic mode of artificial arthroplasty could reduce the risk of adjacent segment lesions, thereby reducing the rate of additional surgery^26^, ^27^. Therefore, the evaluation of kinematic quality parameters may be more important when attempting to predict the early degeneration of surgical segments and adjacent segments. Due to the limitation of research methods, center of rotation and instantaneous axis of rotation are mostly used to replace instantaneous center of rotation postoperatively.

Most studies found that the Bryan disc prosthesis retained the kinematic motion of the cervical spine after arthroplasty. Pickett^28^ measured the position of the center of rotation by dynamic X‐ray of the cervical spine in 20 patients who received Bryan artificial cervical disc implantation at one or two segments before and at intervals up to 24 months after postoperatively. It was found that the center of rotation coordinates of the index and adjacent segments did not change significantly within 24 months after surgery. Ryu *et al.* 20 reported 20 patients who underwent single‐level cervical arthroplasty with a Bryan disc prosthesis. They found that the COR‐X did not change after arthroplasty (1 year postoperatively, *P* = 0.16; 2–3 years postoperatively, *P* = 0.28; and 4–5 years postoperatively, *P* = 0.90). The COR‐Y values also did not change significantly after surgery and remained stable over the course of follow up (1 year postoperative, *P* = 0.13; 2–3 years postoperative, *P* = 0.73; and 4–5 years postoperative, *P* = 0.30). Powell *et al.* 21 reported on 22 patients who underwent Bryan disc replacement. They found that at the arthroplasty level, the COR shifted more posteriorly (0.3 mm, 1% end plate width) and superiorly (4.9 mm, 20% end plate width) compared with the preoperative position; however, this change was not statistically significant (*P* = 0.06). The variability of the COR was lower after arthroplasty than the preoperative values. The Bryan artificial cervical disc provided in vivo functional spinal motion at the operated level, reproducing the preoperative kinematics of the spondylotic disc.

Galbusera *et al.* 29 established a complete finite element model of C_4_–C_7_ and a finite element model of C_5–6_ implanted artificial intervertebral discs (Bryan). It was found that the position of the center of rotation of the complete cervical vertebral model coincided well with the position described by Penning^15^. It was located in the posterior region of the C_6_ vertebral body and moved towards the upper end plate of C_6_ during flexion and backward extension. Comparing the complete cervical spine model with the C_5–6_ artificial disc model, it was found that the center of rotation did not exactly correspond to the operative segment, but there was no significant difference in the movement pattern. It was supposed that a cervical disc prosthesis could maintain motion and yield a near‐physiological center of rotation at the implanted segment. However, this was a study in computer engineering. Factors such as cervical degeneration were not considered.

Other disc prostheses can also retain the quality of cervical motion postoperatively. Kowalczy *et al.* 30 performed a retrospective analysis of 120 X‐ray films of 20 patients after single‐segment PRESTIGE LP implantation was performed. Static and dynamic radiological assessments were performed before and 1 year after the operation. It was found that the PRESTIGE LP maintained preoperative ROM, translation, and COR X values. The postoperative COR Y value changed significantly by shifting superiorly.

Kim *et al.* 31 performed a retrospective analysis of 11 patients undergoing cervical arthroplasty at the C_5–6_ segment. The instantaneous axis of rotation was calculated according to preoperative and 6‐month postoperative dynamic radiographs. It was found that although significant inferior shift occurred at C_6–7_ after TDR (*P* = 0.02), the shift occurred within the normal range in the cervical normogram. There was no significant change in the instantaneous axis of rotation after artificial disc replacement, but the sample number was too small to draw a definite conclusion.

Compared with ACDF, cervical arthroplasty can retain qualitative motion of the cervical spine. Park *et al.* 32 conducted a multicenter and prospective randomized study in 272 patients who underwent cervical arthroplasty surgery and 182 patients who underwent ACDF surgery. The X‐ray images preoperatively and 3, 6, and 12 months postoperatively were evaluated. It was found that the center of rotation moved anteroinferiorly 1.5 mm compared with its position before cervical arthroplasty. This movement was still within the range of the center of rotation of normal subjects, and the center of rotation of the adjacent segments was not affected after cervical arthroplasty. However, the shift in the position of the superior adjacent segments after ACDF increased significantly, accompanied by an increasing trend in translation. Cunningham *et al.* 33 used 8 fresh cervical spine cadavers to conduct a comparative study of single‐segment and double‐segment cervical arthroplasty and ACDF. It was found that the center of rotations of the operative segment (C_6_–C_7_) and the adjacent segment (C_5_–C_6_ and C_7_–T_1_) were located in the center of lower vertebral body in the intact specimens without operation. After single‐segment artificial disc replacement, the center of rotations were evenly distributed in the lower vertebral body of the operated and adjacent segments, and more diffuse center of rotations were produced after single‐segment fusion, compared with those without surgery. It is suggested that the change of center of rotation caused by ACDF may be the potential cause of adjacent segment degeneration^34^, ^35^.

Liu *et al.* 36 performed kinematics analysis on 5 fresh whole cadaver specimens. Compared with single fusion, hybrid surgery (artificial disc replacement + fusion) and 2‐segment disc replacement did not change ROM, and only minimally changed the adjacent segment ICR. Hybrid surgery and cervical arthroplasty retained not only quantitative but also qualitative motion. There is still no high‐quality evidence to compare the efficacy of cervical arthroplasty, ACDF, and hybrid surgery in the treatment of cervical degeneration. However, there is no significant difference in the ROM and COR changes in adjacent segments after cervical arthroplasty and ACDF^23^.

In summary, most studies consider that there is no significant difference in the position of quality parameters between the operative segment and adjacent segment after cervical arthroplasty, and there is no effect on the kinematics of the operative segment and adjacent segment. After some prosthetic replacements, there is a change in the vertical direction of the operative segment, but the adjacent segment is basically retained. The change in the kinematics environment after fusion is considered to be the main contributing factor to the degeneration of adjacent segments. However, there is still controversy regarding the changes in kinematic quality parameters after different surgical procedures, which needs further confirmation with large‐sample clinical studies. Compared with ACDF, cervical arthroplasty has the advantage of maintaining the quality of motion, and it has a minor impact on adjacent segment degeneration. However, Rousseau *et al.* 25 investigated the intervertebral sagittal ROM and COR in 26 patients with the Prestige LP prosthesis (Medtronic Sofamor Danek, Memphis, TN, USA) and 25 patients with the Prodisc‐C prosthesis (Synthes, West Chester, PA, USA), comparing them with the measurements of 200 healthy cervical discs in vivo, and found that although the COR‐FE remained within the normal range in most cases, it tended to be located more anteriorly and superiorly than normal in patients with the 2 types of prostheses. Neither the cranial nor caudal types of ball‐and‐socket designs fully restored normal mobility in terms of ROM and center of rotation in this patient series.

Therefore, kinematic quality parameters are effective and important parameters for evaluating changes in the cervical motion pattern after cervical fusion and nonfusion. They can be used to evaluate the effects of different surgical methods on operative and adjacent segments in the early stages and predict the occurrence of adjacent segment degeneration. However, due to the limitation of current research methods, the current research is basically limited to flexion and extension of the cervical spine, and further studies on compound movement are needed.

Although cervical arthroplasty has an advantage over ACDF in retaining cervical motion quality, the implant position and type of prosthesis in cervical arthroplasty impact cervical motion quality postoperatively^41^. Different rotation centers in the prevalent artificial cervical discs produce different biomechanical performances^43^.

Kowalczyk *et al.* 37 retrospectively analyzed 60 patients who had undergone Bryan, ProDisc‐C, and Prestige LP prosthesis implantations. Quantitative measurement analysis software was used to evaluate cervical spine X‐ray images to determine the COR locations preoperatively and 1 year postoperatively. Significant differences were found in the COR‐X and COR‐Y parameters between the Bryan and ProDisc‐C and between the Bryan and Prestige LP prostheses. The Bryan prosthesis did not significantly vary in the COR‐X or COR‐Y; the COR‐X of ProDisc‐C moved significantly anterior, and the COR‐Y did not vary significantly. After Prestige LP implantation, the COR‐X remained unvaried, while the COR‐Y moved significantly superiorly. The Bryan disc best preserved the physiological location of the preoperative COR.

Patwardhan *et al.* 38 used 12 human cadaveric cervical spines (C_3_–C_7_) and performed cervical arthroplasty in the C_5_–C_6_ segment using M6 prosthesis. When performing a flexion–extension movement, the center of rotation did not deviate significantly from that of intact cervical cadaveric specimens when the prosthesis was implanted in the middle position. When the prosthesis was implanted in the posterior position, the center of rotation moved significantly more posteriorly than the location in the intact cervical cadaveric specimen.

Lee^39^ used finite element simulation to analyze the variations of the center of rotation after three different artificial intervertebral disc (ProDisc‐C, Discocerv, Baguera C) replacements (C_5–6_ segment). Only Baguera C was found to simulate the center of rotation locations of the intact cervical spine model during flexion. However, this experiment was a simulation of the nonphysiological state, and the center of rotation analysis therefore had limitations.

Barrey *et al.* 40 studied the changes of center of rotation in 32 patients undergoing C_5–6_ artificial disc replacement and confirmed that the position of the center of rotation in vivo was highly correlated with the design of the artificial disc, especially with the position of the center of rotation in the prosthesis.

Therefore, to protect the facet joints from abnormal forces and pressures, to retain the quality of motion, and to avoid degeneration of adjacent segments, cervical artificial disc prosthesis should try to maintain the normal cervical instantaneous center of rotation, simulate cervical physiological movement, and restore sagittal balance. Selecting a suitable prosthesis, considering the kinematic quality parameters of the device, especially the position of the instantaneous center of rotation, and designing the artificial disc individually are the prerequisites for fully realizing the goal of cervical arthroplasty to retain motion and achieve non‐fusion.

## Clinical Application of Kinematic Quality Parameters in Cervical Deformity Correction

Studies on parameter locations in cervical deformities are lacking, and they have not been fully applied in clinical preoperative evaluation and surgical planning. By understanding the location of instantaneous axis of rotation, the arm length of the correction force can be determined and the degree of biomechanical advantage can be determined^11^. If the structure changes or is damaged, the instantaneous axis of rotation will migrate to the most dense or hardest part of the structure. Through understanding the changes in the center of rotation of thoracic kyphosis osteotomy after Ponte osteotomy, it is evident that the center of rotation of thoracic kyphosis deformity moves to the front of the spine after Ponte osteotomy, which will lead to the elongation of the force arm of the posterior correction deformity, and produce positive biomechanical advantages.^42^ Through the change in the center of rotation after thoracic deformity osteotomy, we know that three important factors should be addressed when spinal deformity osteotomy is performed, including the correction segment, the degree of stiffness, and positions of the target quality parameters of the correction, which must be considered to achieve correction, so as to guide the operation of cervical vertebral deformity osteotomy. Previous studies have confirmed that different osteotomy methods of cervical deformity will have different effects on the cervical motion pattern and the position of the quality parameters. Choosing the correct surgical method to achieve the best surgical effect is still a challenge, Koller *et al.* 6 studied the change of center of rotation among 23 patients with AS: 11 patients received a PSO and 12 an SPO. The author found that COR‐X was 2.7% ± 49.6% and COR‐Y was 25.5% ± 55.2%. They confirmed that the actual osteotomy center of rotation for PSO, SPO, and YTO were far from the conceptional center of rotation. Determining how to obtain a satisfactory position of center of rotation after surgery is still a major challenge for cervical spine osteotomy. How to obtain a satisfactory position of the center of rotation postoperatively is still a major challenge in cervical spine osteotomy. The osteotomy COR was calculated from the intersection of perpendicular bisectors that connect to identical landmarks on anterior and posterior vertebral body elements on preoperative and postoperative CT images.^6^. For cervical deformity correction, the center of rotation should be reconstructed after osteotomy and located at spinal cord level, as this would neither distract nor shorten the cord during osteotomy closure. For patients with different types of cervical deformity, according to the change in the position of kinematic quality parameters under the condition of disease, combined with the improvement of the position of the quality parameters by orthopaedic surgery, a more comprehensive preoperative evaluation can be carried out, and the best operative technique can be selected individually, which can effectively reduce the cost of treatment. The best postoperative effect can also be obtained. Therefore, the location of kinematic quality parameters in cervical deformity and the evaluation of their surgical effect will be a hot research topic in the future.

### *Conclusion*

This review analyzed the published literature relating to the clinical relevance of cervical kinematics. The present study highlights the instantaneous center of rotation, the center of rotation, and the instantaneous axis of rotation, which are important quality parameters of cervical spinal motion. Changes in kinematic parameters have been associated with clinical symptoms. The results presented also showed that kinematic quality parameters can be used to detect abnormal cervical mobility, to assist in the diagnosis of cervical degeneration, and to evaluate surgical effects. These parameters could be used to classify cervical diseases. However, no uniform standard exists for the location of cervical kinematic quality parameters in normal asymptomatic, degenerative, and postoperative subjects. Further study is required to examine these parameters.
